# Lower corticospinal excitability and greater fatigue among people with multiple sclerosis experiencing pain

**DOI:** 10.1177/20552173221143398

**Published:** 2023-01-05

**Authors:** Hannah M. Murphy, Christopher M. Fetter, Nicholas J. Snow, Arthur R. Chaves, Matthew B. Downer, Michelle Ploughman

**Affiliations:** Recovery & Performance Laboratory, Faculty of Medicine, Memorial University of Newfoundland, St John's, Newfoundland and Labrador, Canada

**Keywords:** Corticospinal excitability, pain, multiple sclerosis, transcranial magnetic stimulation, motor evoked potential

## Abstract

**Introduction:**

Persons with multiple sclerosis (MS) frequently report pain that negatively affects their quality of life. Evidence linking pain and corticospinal excitability in MS is sparse. We aimed to (1) examine differences in corticospinal excitability in MS participants with and without pain and (2) explore predictors of pain.

**Methods:**

Sixty-four participants rated their pain severity on a visual analog scale (VAS). Transcranial magnetic stimulation (TMS) and validated clinical instruments characterized corticospinal excitability and subjective disease features like mood and fatigue. We retrieved information on participants' prescriptions and disability status from their clinical records.

**Results:**

Fifty-five percent of participants reported pain that affected their daily functioning. Persons with pain had significantly greater fatigue and lower area under the excitatory motor evoked potential (MEP) recruitment curve (eREC AUC), a measure of total corticospinal excitability. After controlling for age, disability status, and pain medications, increased fatigue and decreased eREC AUC together explained 40% of the variance in pain.

**Discussion:**

Pain in MS is multifactorial and relates to both greater fatigue and lesser corticospinal excitability. Future work should better characterize relationships between these outcomes to develop targeted pain interventions such as neuromodulation.

**Summary:**

We examined pain in MS. Individuals with pain had higher fatigue and lower corticospinal excitability than those without pain. These outcomes significantly predicted self-reported pain.

## Introduction

Pain affects more than 60% of people with multiple sclerosis (MS).^1^ Pain is associated with increased depression, anxiety, cognitive impairment, and fatigue,^2–4^ and compromises employment activities, social engagement, and independent functioning to reduce the quality of life.^3–6^ Despite the deleterious impact of pain, therapies are often inadequate.^5^

MS pain arises from various sources that have distinct pathophysiology and treatment targets.^5–8^ Neuropathic pain is a common and disabling form of MS-related pain,^5,6^ and occurs with lesions affecting primary afferents, the spinothalamic tract or spino-thalamo-cortical network, deep gray nuclei, optic nerves, brainstem, or cranial nerve V/IX/X nuclei.^8,9^ Neuropathic pain therapies target central sensitization by modulating glutamate and gamma-aminobutyric acid (GABA).^10^

The corticospinal tract conveys motor activity from the primary motor cortex to the peripheral nervous system. Integration of sensory and motor signals is critical for goal-directed behaviors.^11^ Using transcranial magnetic stimulation (TMS), experimenters can characterize—and modulate—pain- and sensory-related functional brain networks that involve the primary motor cortex.^12,13^ Neuropathic pain is associated with diminished GABAergic corticospinal inhibition, which correlates with greater pain severity and poorer quality of life.^14^ In MS pain, TMS investigations are sparse. One study found no significant difference in resting motor threshold (RMT) or motor evoked potential (MEP) amplitude between persons with and without neuropathic pain; no significant change in RMT or MEP amplitude after a 1-month sublingual cannabis extract (Sativex^®^) intervention, despite improved pain and spasticity; and no significant correlation between pain, and RMT or MEP amplitude.^15^ Large effect sizes of group differences in RMT and MEP amplitude suggested the study was underpowered.^15^

Given the paucity of TMS literature in MS-related pain, we aimed to (1) examine differences in corticospinal excitability or inhibition in MS participants with versus without pain and (2) explore predictors of MS pain. We hypothesized that reduced corticospinal inhibition would be associated with greater pain severity, and that corticospinal disinhibition would significantly predict higher pain, after controlling for disability status and pain medications.

## Materials and methods

### Study design

We conducted a cross-sectional study of an MS cohort in Newfoundland and Labrador, Canada, from 2016 to 2019. Participants completed the study at a tertiary rehabilitation hospital-based neurorehabilitation research laboratory, on a single 3-h occasion. We collected demographic and disease information and self-reports of pain, fatigue, depression, and anxiety. We performed a standardized screening assessment for mild cognitive impairment and TMS assessments of corticospinal excitability and inhibition. We retrieved information on prescriptions (disease-modifying therapy [DMT], analgesics, neuropathic pain medications) and disability status from clinic records. Participants gave written informed consent per the Declaration of Helsinki. The Human Research Ethics Board approved the study (HREB Ref: 2015.103).

Previous cross-sectional work showed a large Cohen's *d* effect size^16^ of ∼2 when comparing changes in MEP amplitude between MS participants with versus without neuropathic pain, following a 1-month cannabis extract intervention.^15^ Using *d* = 2, α = 0.05, and power (1 – β) = 0.80, for a two-tailed Mann–Whitney *U* test with 1:1 allocation and a log-normal distribution, we required 24 participants (*n* = 12 per group). When we considered MS research using regression analysis for clinical predictors, we estimated a final required sample (*n*) of 60.^17^

### Participants

We included participants who (1) were ≥18 years old, (2) diagnosed using Revised McDonald Criteria,^18^ and (3) completed a pain visual analog scale (VAS). We excluded participants with (1) contraindications to TMS,^19^ (2) active disease in the last 3 months, or (3) non-participation in TMS testing. We extracted participants' disease characteristics from health records (i.e., disease duration [years], disease course [relapsing, progressive], and disability status [Expanded Disability Status Scale, EDSS]).^20^

### Questionnaires

Participants reported their pain severity using a 100 mm VAS asking, “Today, how much does pain affect your daily life and relationships?” Pain ≥4 mm was clinically significant, because of its negative effect on daily functioning,^21^ and was used to distinguish groups with pain versus no pain. The VAS is recommended for pain measurement in MS because of its greater sensitivity than other instruments.^22^ In non-MS studies of postsurgical, chronic musculoskeletal, and neuropathic pain, the VAS has moderate reliability and construct validity.^21,23^ The pain VAS is reliable in MS research.^24^

We assessed fatigue using a 100 mm VAS.^25^ We used ≥4 mm to distinguish clinically significant fatigue.^21^ In MS research, the fatigue VAS is reliable,^24^ has moderate construct validity,^25,26^ and is up to 76% sensitive and 72% specific for clinically significant fatigue.^25^

We used the Hospital Anxiety and Depression Scale (HADS) to measure depression and anxiety symptoms. This 14-item instrument, with seven questions each pertaining to anxiety and depression, is validated in MS.^27^ We used depression and anxiety scores ≥8 and 7 points to identify depression and anxiety. While others suggest a threshold score ≥11 points can detect clinically significant depression and anxiety in MS,^28^ the initial validation study in MS used a larger and more representative sample of 320 (vs. 34) participants. That study found cutoffs ≥8 and 7 points had optimal sensitivity (90%, 78%) and specificity (87%, 78%) for major depression and generalized anxiety, respectively.^27^ The HADS is reliable and has good incremental-, concurrent-, and construct validity in MS.^29^

We screened for mild cognitive impairment using the Montreal Cognitive Assessment (MoCA), a 30-point tool that investigates visuospatial abilities, language, attention, memory, abstraction, and orientation, with scores <26 suggestive of mild cognitive impairment.^30^ The MoCA has good construct validity, moderate reliability, and up to 91% sensitivity and 93% specificity for mild cognitive impairment in MS.^31,32^

### TMS data collection

We used single-pulse TMS to assess corticospinal excitability and inhibition.^33^ A BiStim 2002 stimulator with a 70 mm figure-of-eight coil (Magstim Co., Whitland, UK) delivered monophasic pulses to elicit MEPs from the weaker first dorsal interosseous (FDI). Interhemispheric differences in TMS measures from the weaker versus stronger sides in MS mirror hand dominance in healthy controls.^33–35^ Neuropathic pain in MS is associated with corresponding limb weakness,^36^ and the painful weaker limb in other pain research shows corresponding corticospinal excitability abnormalities.^37^ In MS, the hemisphere of the weaker hand has lower corticospinal excitability and higher corticospinal inhibition than the stronger side.^33–35^ Observations from the weaker side of persons with MS are also better correlated with hand dexterity, walking performance, cognitive processing speed, fatigue, disability status, and heat sensitivity.^4,37,38^ We determined the weaker hand using calibrated pinch and handgrip dynamometers (B&L Engineering, Santa Ana, CA, USA). Surface electrodes with a belly-tendon montage (Kendall 200, Covidien, Mansfield, MA) transmitted electromyographic (EMG) activity to the recording device (Brainsight^™^, Rogue Research, Montreal, QC, Canada; 2500 V/V amplification, 3 kHz sampling, 600 V/V gain, 5–550 Hz bandwidth). We sampled EMG from 100 ms pre-stimulus to 800 ms post-stimulus to capture both MEPs and contralateral corticospinal silent periods (CSP). We also recorded participants' self-reported hand dominance.

We used Brainsight^™^ (Rogue Research, Montreal, QC, Canada) to define the hotspot and guide coil placement. We stimulated over the precentral gyrus of the hemisphere contralateral to the weaker hand, with the coil handle oriented posterolaterally at a 45° angle to the midsagittal line, to induce a posterior–anterior current in the underlying cortex.^39,40^ The hotspot was the point with the greatest MEP amplitude in FDI from suprathreshold stimulation during a 10% maximal tonic contraction. We defined active motor threshold (AMT) as the minimum TMS intensity to elicit MEPs ≥200 µV, from ≥5 of 10 trials, during a 10% maximal tonic contraction of FDI.^40^ AMT assesses glutamate-mediated excitability of low-threshold neurons and reflects the bias level of the corticospinal representation.^39,40^ Abnormal values reflect demyelination and axonal damage.^41^

We next collected MEP and CSP data. During a 10% maximal tonic contraction of weaker FDI, we elicited MEPs from the hemisphere contralateral to the weaker hand. We delivered six pulses each, between 105% and 155% AMT, in randomized blocks of 10% AMT. We used a randomized 4- to 10-s interpulse interval and allowed participants a brief rest between blocks. We produced excitatory (eREC) and inhibitory (iREC) MEP recruitment curves, based on MEP amplitude (µV) and CSP duration (ms) versus stimulus intensity (% AMT). MEP recruitment curves characterize the input–output properties of corticospinal motor neurons.^39,40^ MEP amplitude reflects voltage-gated ion channels and glutamate activity in corticospinal pyramidal neurons.^39,40^ CSP duration characterizes the activity of inhibitory GABAergic interneurons.^39,40^ We also used MEP latency to characterize corticospinal conduction.^39–41^

### TMS data processing

We processed TMS data using Signal software v6.04 (Cambridge Electronic Design, Cambridge, UK). We inspected MEPs for artifact exceeding 100 µV, omitting <1% of trials. We reported AMT as a percentage of maximal stimulator output (% MSO),^39,40^ MEP amplitude as peak-to-peak amplitude (µV),^39,40^ MEP latency as the height-normalized time (ms/cm) from TMS pulse to MEP onset (EMG amplitude >2 standard deviations [SD] of mean background activity),^39,40^ and CSP duration as the time (ms) from MEP onset to the return of EMG to background activity, normalized to MEP amplitude.^39,40^ We used a quantitative graphical method, with the active cursor feature of Signal, to identify MEP onset and CSP offset.^38^ To resolve eREC and iREC, we plotted MEP amplitude (µV) and CSP (ms/µV) against stimulus intensity (% AMT).^39,40^ We calculated the area under the recruitment curves (AUC), which characterizes overall corticospinal output^42^ and correlates with MS functional outcomes.^33^

## Statistical analysis

We performed statistical analysis using IBM SPSS v.27 (Armonk, NY, USA). We inspected data distributions using Shapiro–Wilk tests.^43^ Tests were two-tailed, with a statistical significance threshold of *p* < .05, unless otherwise stated. We reported effect sizes with 95% confidence intervals (95% CI).^44^

### Groupwise comparisons

We used Mann–Whitney *U* tests (continuous variables) and chi-square (*χ*^2^) tests (categorical variables) to compare demographic and disease information (sex, age, MS type, disease duration, EDSS, medications), questionnaires (fatigue VAS, HADS, MoCA), and TMS outcomes (AMT, MEP latency, eREC AUC, iREC AUC) between persons with and without pain.^44^ For continuous variables, we reported median (*M*) and interquartile range (IQR). For categorical variables, we reported proportion and percentage. *U* test effect sizes (*r*) were trivial if <0.1, small if 0.1–0.3, medium if 0.3–0.5, and large if >0.5.^16^ *χ*^2^ test effect sizes (*h*) were trivial if <0.2, small if 0.2–0.5, medium if 0.5–0.8, and large if >0.8.^16^

### TMS–clinical associations

To explore associations between clinical and TMS data in each group, we performed separate bivariate correlations (Spearman's rho [ρ] for continuous–continuous, Kendall's tau-b [τ] for continuous–categorical).^44^ Because of the large number of correlations, we used the Bonferroni correction to adjust the statistical significance threshold (*p* < .05 divided by 34 tests = *p* < .0015).^43^ We compared statistically significant correlations across groups using Fisher *z*-transformations and effect sizes (Cohen's *q*) with 95% CIs.^16^ Correlations (ρ, τ) were zero if <0.1, weak if 0.10–0.39, moderate if 0.40–0.69, strong if 0.70–0.99, and perfect if 1.0.^45^ Effect sizes (*q*) were trivial if <0.2, small if 0.2–0.5, medium if 0.5–0.8, and large if >0.8.^16^

### Predictors of pain

To determine whether corticospinal excitability or inhibition and/or clinical MS features predicted pain (yes, no), we entered outcome measures that differed significantly between groups as predictors into a binomial logistic regression, controlling for age, EDSS, and neuropathic pain and analgesic medications (yes, no).^44,46^ We added predictor variables in separate blocks, ordered by effect size. We established independence of observations, lack of multicollinearity and outliers, and linear relationships between predictors and the logit of the dependent variable.^47^ Box–Tidwell and Hosmer–Lemeshow tests assessed linearity and goodness of fit.^47^ We reported sensitivity, specificity, percentage accuracy of classification, and predictive values. Nagelkerke *R*^2^ was very weak if <0.3, weak if 0.3–0.5, moderate if 0.5–0.7, or strong if >0.7.^48^

## Results

### Participants

**Figure 1.**
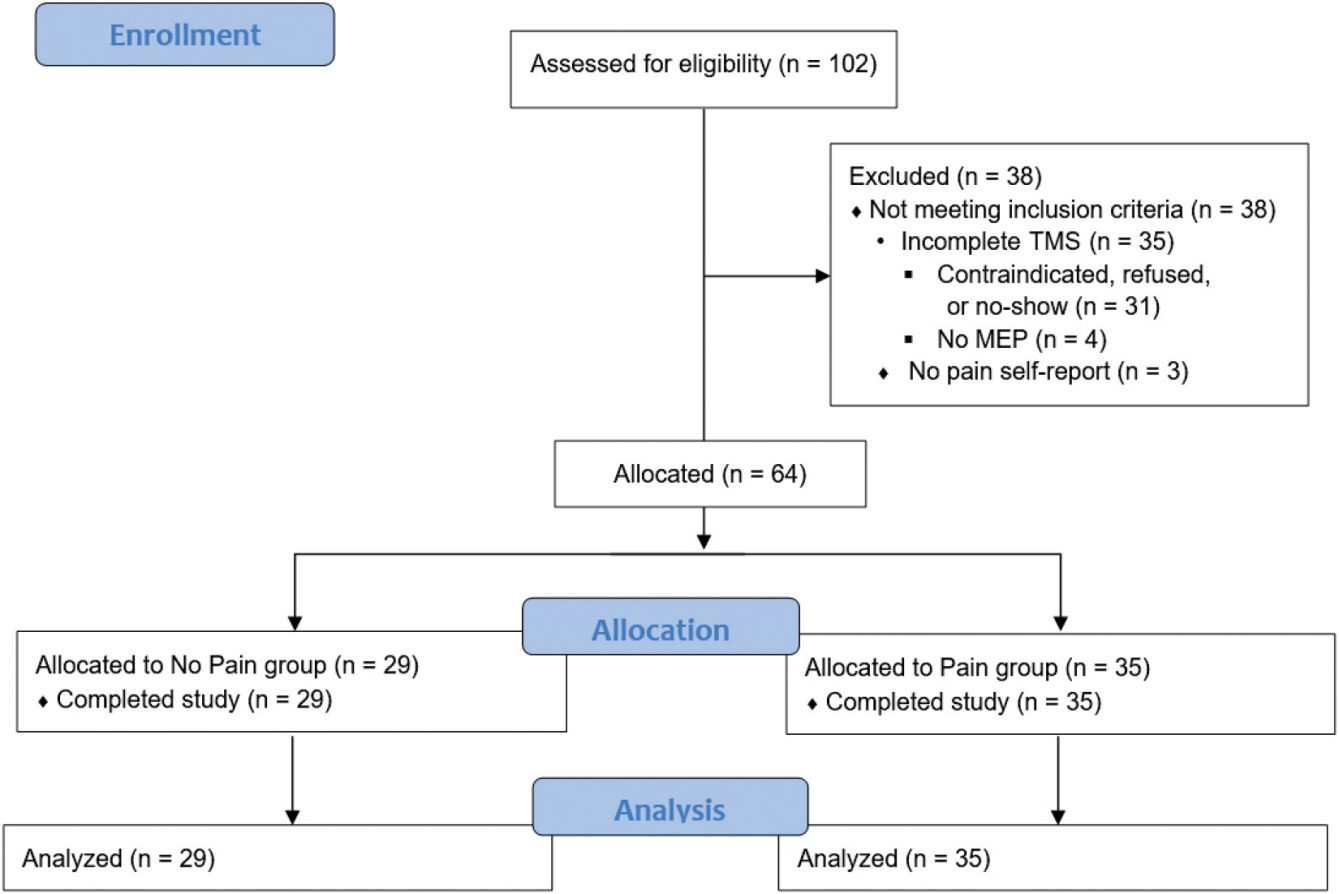
Flowchart of participant enrollment, allocation, and analysis. We recruited 102 consecutive participants volunteers from a local MS clinic who agreed to take part in a longitudinal MS cohort study. Inclusion criteria were: (1) diagnosis of MS and (2) age 18 years of age or older. We excluded participants with (1) absolute contraindications to TMS, (2) history of active disease or systemic corticosteroid use in the last 3 months, (3) no pain self-report, or (4) incomplete TMS testing which prevented data analysis. We excluded three individuals who did not complete pain self-reports. Another 35 did not complete TMS testing, preventing data analysis. We included 64 participants for data analysis. Twenty-nine participants (45%) did not report having pain (no pain group), and 35 (55%) reported pain that had a functional impact on their life (pain group).

We screened 102 participants for eligibility (Figure 1). We excluded three individuals who did not complete the pain VAS and 35 who did not complete TMS testing. Sixty-four participants, 29 of whom reported no pain (45%) and 35 who reported clinically significant pain (55%), completed the study (Table 1).

**Table 1.** Demographic and disease information.

Variable	All participants (*n* = 64)		No pain (*n* = 29)		Pain (*n* = 35)		No pain vs. pain					
	*M* or *n*	IQR or %	*M* or *n*	IQR or %	*M* or *n*	IQR or %	*U* or *χ*^2^	*p*-value	*r* or *h*	95% CI lower bound	95% CI upper bound	Effect size
Age (years)	47	15	44	16	51	13	653.0	.050	0.25	−0.01	0.50	Small
Sex (F, M)	F = 46	F = 72%	F = 20	F = 69%	F = 26	F = 74%	0.222	.637	−0.08	−0.50	0.35	Trivial
	M = 18	M = 28%	M = 9	M = 31%	M = 9	M = 26%						
EDSS (0–10)	2	1	2	2	2	1.5	586.5	.274	0.14	−0.11	0.39	Small
Disease duration (years)	15	13	14	14	15	12	606.0	.184	0.17	−0.08	0.42	Small
MS type (RMS, SPMS, SPMS)	RMS = 58	RMS = 90%	RMS = 27	RMS = 93%	RMS = 31	RMS = 89%	0.921	.631	0.11	−0.11	0.11	Trivial
	SPMS = 5	SPMS = 8%	SPMS = 2	SPMS = 7%	SPMS = 3	SPMS = 9%						
	PPMS = 1	PPMS = 2%	PPMS = 0	PPMS = 0%	PPMS = 1	PPMS = 2%						
Handedness (L, R)	L = 6	L = 9%	L = 3	L = 10%	L = 3	L = 9%	0.059	.809	−0.04	−0.24	0.16	Trivial
	R = 59	R = 91%	R = 26	R = 90%	R = 32	R = 91%						
Weaker hand (L, R)	L = 49	L = 77%	L = 21	L = 72%	L = 28	L = 80%	0.509	.476	−0.12	−0.50	0.26	Trivial
	R = 15	R = 23%	R = 8	R = 28%	R = 7	R = 20%						
Weaker dominant hand (Y, N)	Y = 19	Y = 30%	Y = 11	Y = 38%	Y = 8	Y = 23%	1.726	.189	0.09	−0.37	0.54	Trivial
	N = 45	N = 70%	N = 18	N = 62%	N = 27	N = 77%						
DMT (Y, N)	Y = 48	Y = 75%	Y = 25	Y = 86%	Y = 23	Y = 66%	3.552	.059	0.32	−0.13	0.77	Small
	N = 16	N = 25%	N = 4	N = 14%	N = 12	N = 34%						
Analgesics (Y, N)	Y = 56	Y = 88%	Y = 26	Y = 90%	Y = 30	Y = 86%	0.225	.635	0.08	−0.24	0.41	Trivial
	N = 8	N = 12%	N = 3	N = 10%	N = 5	N = 14%						
Neuropathic pain medication (Y, N)	Y = 45	Y = 70%	Y = 23	Y = 79%	Y = 22	Y = 63%	2.057	.152	0.24	−0.22	0.69	Small
	N = 19	N = 30%	N = 6	N = 21%	N = 13	N = 37%						

95% CI: 95% confidence interval; DMT: disease-modifying therapy; EDSS: Expanded Disability Status Scale; IQR: interquartile range; L: left; *M*: median; MS: multiple sclerosis; N: no; PPMS: primary progressive MS; R: right; RMS: relapsing MS; SPMS: secondary progressive MS; Y: yes.

Seventy-two percent of participants were female, in line with previous observations.^3–6^ Most participants had relapsing MS, low EDSS (*M* = 2), and DMT prescriptions. Participants with pain were older, but this difference was small and borderline significant (*p* = .050, *r* = 0.25). More pain-free participants were prescribed DMT, but the difference was small and borderline significant (*p* = .059, *h* = 0.32). Participant characteristics were otherwise not significantly different (*p* > .05).

### Greater fatigue in persons with pain

Significantly more participants with pain reported clinically significant fatigue (*p* = .005, *h* = 0.25; Table 2). The magnitude of difference was borderline significant (*p* = .066, *r* = 0.23). There were no significant differences in anxiety, depression, or cognitive impairment (*p* > .05).

**Table 2.** Comparison of questionnaire data between participants with and without pain.

Variable	All participants (*n* = 64)		No pain (*n* = 29)		Pain (*n* = 35)		No pain vs. pain					
	*M* or *n*	IQR or %	*M* or *n*	IQR or %	*M* or *n*	IQR or %	*U* or *χ*^2^	*p*-value*	*r* or *h*	95% CI lower bound	95% CI upper bound	Effect size
Fatigue												
VAS (0–100 mm)	42.5	49.3	25.0	59.0	49.0	43.0	643.5	.066	0.23	0.02	0.48	Small
Y, N (≥4 mm)	Y = 55	Y = 86%	Y = 21	Y = 72%	Y = 34	Y = 97%	8.025	.005*	0.25	0.13	0.63	Small
	N = 9	N = 14%	N = 8	N = 28%	N = 1	N = 3%						
Anxiety^a^												
HADS (0–21 points)	7.0	6.0	7.0	5.8	5.0	6.5	318.5	.996	−0.07	−0.32	0.18	Trivial
Y, N (≥7 points)	Y = 21	Y = 39%	Y = 9	Y = 37%	Y = 12	Y = 41%	0.083	.774	0.05	−0.45	0.55	Trivial
	N = 32	N = 61%	N = 15	N = 63%	N = 17	N = 59%						
Depression^a^												
HADS (0–21 points)	3.0	5.5	2.0	6.0	3.0	5.0	374.0	.751	0.06	−0.19	0.31	Trivial
Y, N (≥8 points)	Y = 11	Y = 21%	Y = 5	Y = 21%	Y = 6	Y = 21%	0.0002	.996	−0.002	−0.29	0.29	Trivial
	N = 42	N = 79%	N = 19	N = 79%	N = 23	N = 79%						
Cognitive impairment												
MoCA (0–30 points)	27.0	2.8	27.0	2.0	27.0	3.0	500.0	.681	−0.01	−0.26	0.24	Trivial
Y, N (<26 points)	Y = 13	Y = 20%	Y = 5	Y = 17%	Y = 8	Y = 23%	0.309	.578	0.10	−0.15	0.34	Trivial
	N = 51	N = 80%	N = 24	N = 83%	N = 27	N = 77%						

^a^ We analyzed anxiety and depression in a subsample of 53 participants (*n* = 11 missing). Positive symptom screens are from threshold scores on Fatigue visual analog scale (VAS), Hospital Anxiety and Depression Scale (HADS), and Montreal Cognitive Assessment (MoCA). 95% CI: 95% confidence interval; N: no; Y: yes.

*Statistically significant at *p* < .05.

### Lower total corticospinal excitability in persons with pain

**Figure 2.**
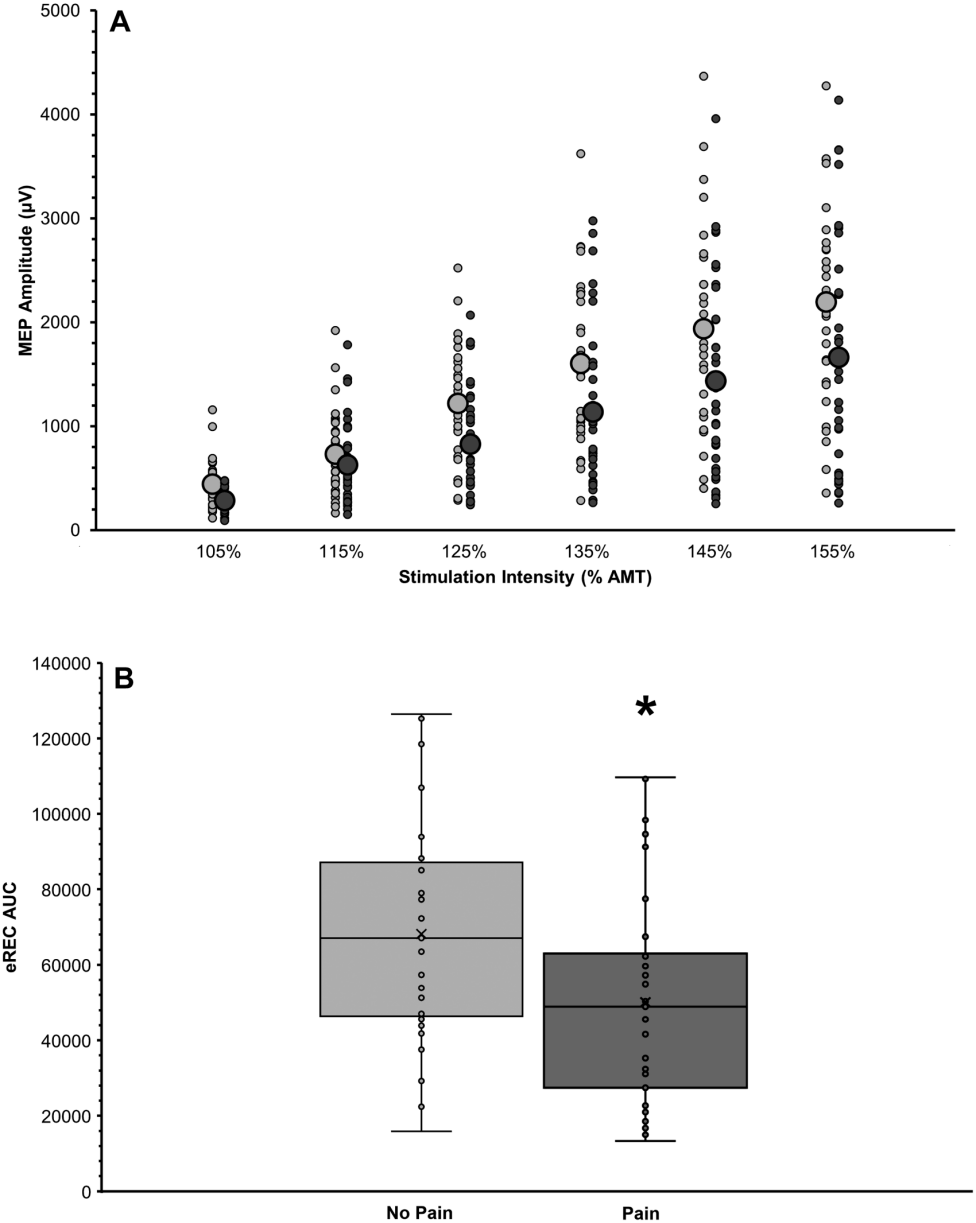
Comparison of total corticospinal excitability (excitatory motor evoked potential [MEP] recruitment curve, eREC) in participants with (dark gray) and without pain (light gray). A. MEP recruitment curves exhibiting TMS stimulator intensity (percentage of active motor threshold [% AMT]; *x*-axis) raw MEP amplitude (µV; *y*-axis). Plot shows individual data points with larger circles depicting the group means. B. Boxplot showing groupwise comparison of eREC area under the curve (AUC). Plots include individual data points, means (×) and medians (horizontal line), interquartile ranges (boxes), and 95% confidence intervals (error bars). A Mann–Whitney *U* test on eREC AUC values showed that persons without pain had significantly greater total corticospinal excitability (*p* = .019), although the effect size was small (*r* = 0.29).

Participants with pain exhibited significantly lower total corticospinal excitability (eREC AUC; *p* = .019, *r* = −0.29; Figure 2) but no significant differences in other TMS variables (AMT, MEP latency, iREC AUC; *p* > .05; Table 3).

**Table 3.** Comparison of transcranial magnetic stimulation (TMS) data between participants with and without pain.

Variable	All participants (*n* = 64)		No pain (*n* = 29)		Pain (*n* = 35)		No pain vs. pain					
	*M*	IQR	*M*	IQR	*M*	IQR	*U*	*p*-value*	*r*	95% CI lower bound	95% CI upper bound	Effect size
AMT (% MSO)	33	13	32	11	35	16	572.0	.384	0.11	−0.14	0.36	Small
MEP latency (ms/cm)	0.137	0.015	0.137	0.016	0.136	0.015	537.0	.691	0.05	−0.20	0.30	Trivial
eREC AUC	54,736.24	42,519.14	67,078.55	40,865.13	48,900.83	35,614.08	333.0	.019*	−0.29	−0.55	−0.04	Small
iREC AUC	4.44	3.91	4.44	3.12	4.44	4.92	622.0	.123	0.17	−0.08	0.42	Small

95% CI: 95% confidence interval; % MSO: percentage of maximum TMS stimulator output; AMT: active motor threshold; AUC: area under the curve; eREC: excitatory motor evoked potential (MEP) recruitment curve; iREC: inhibitory MEP recruitment curve; IQR: interquartile range; *M*: median.

*Statistically significant at *p* < .05.

### Clinical–TMS associations

We found moderate associations between more progressive MS and higher EDSS, higher AMT, prolonged MEP latency, and lower eREC AUC in participants with pain (ρ/τ = 0.50–0.56, *p* < .001; Table 4). In those without pain, there was a moderate correlation between higher EDSS and more neuropathic pain medication prescriptions (τ = −0.55, *p* = .001).

**Table 4.** Simple bivariate correlations (Spearman's rho, ρ) showing clinical–TMS associations.

Correlation	No pain (*n* = 29)		Pain (*n* = 35)		No pain vs. pain					
	ρ, τ	*p*-value**	ρ, τ	*p*-value**	*z*	*p*-value**	*q*	95% CI lower bound	95% CI upper bound	Effect size
MS type (RMS, PrMS)—EDSS (0–10)	0.25	.137	0.56	<.001**	1.429	.153	−0.38	−0.63	−0.13	Small
MS type (RMS, PrMS)—AMT (% MSO)	0.27	.094	0.50	<.001**	1.032	.302	−0.36	−0.52	−0.02	Small
MS type (RMS, PrMS)—MEP latency (ms/cm)	0.21	.189	0.52	<.001**	1.375	.169	−0.36	−0.61	−0.11	Small
MS type (RMS, PrMS)—eREC AUC	−0.26	.094	−0.52	<.001**	1.175	.240	−0.31	−0.56	−0.06	Small
EDSS (0–10)—AMT (% MSO)	0.39	.037	0.56	<.001**	0.859	.39	−0.23	−0.48	0.02	Small
EDSS (0–10)—neuropathic pain medications (Y, N)	−0.55	.001**	−0.18	.228	1.653	.098	0.44	0.19	0.69	Small

95% CI: 95% confidence interval; % MSO: percentage of maximum TMS stimulator output; AMT: active motor threshold; EDSS: Expanded Disability Status Scale; eREC AUC: area under the excitatory motor evoked potential recruitment curve; MEP: motor evoked potential; RMS: relapsing multiple sclerosis; MS: multiple sclerosis; N: no; PrMS: progressive (primary and secondary) multiple sclerosis; Y: yes. We adjusted the statistical significance threshold using the Bonferroni correction. We report here only statistically significant associations.

**Statistically significant at *p* < .0015.

### Predictors of pain

In the logistic regression, the control variables (Block 1: age, EDSS, analgesic and neuropathic pain medications) predicted 11.3% of pain variance but did not reach statistical significance (*p* = .226; Table 5). The addition of fatigue (yes, no) in Block 2 explained an additional 14.1% of the variance (Nagelkerke *R*^2^ = 0.254, *p* = .005). In the overall model, eREC AUC (Block 3) explained a further 15.5% of the variance (Nagelkerke *R*^2^ = 0.399, (*p* < .001).

**Table 5.** Binary logistic regression showing predictors of pain (yes or no).

Block	Variables	Nagelkerke *R*^2^	Size of *R*^2^	Sn	Sp	PPV	NPV	PAC	*χ* ^2^	*df*	*p*-value
1	Age (years)	0.113	Very weak	55.2%	71.4%	61.5%	65.8%	64.1%	5.661	4	.226
	EDSS (0–10)										
	Analgesic medications (Y, N)										
	Neuropathic pain medications (Y, N)										
2	Age (years)	0.254	Very weak	48.3%	91.4%	82.4%	68.1%	71.9%	13.464	5	.019*
	EDSS (0–10)										
	Analgesic medications (Y, N)										
	Neuropathic pain medications (Y, N)										
	Fatigue (Y, N)										
3	Age (years)	0.399	Weak	65.5%	80.0%	73.7%	73.1%	73.4%	22.669	6	<.001**
	EDSS (0–10)										
	Analgesic medications (Y, N)										
	Neuropathic pain medications (Y, N)										
	Fatigue (Y, N)										
	eREC AUC										

EDSS: Expanded Disability Status Scale; eREC AUC: area under excitatory motor evoked potential (MEP) recruitment curve; N: no; NPV: negative predictive value; PAC: percentage accuracy of classification; PPV: positive predictive value; Sn: sensitivity; Sp: specificity; Y: yes.

*Statistically significant at *p* < .05. **Statistically significant at *p* < .001.

## Discussion

We set out to determine how pain in MS relates to corticospinal excitability or inhibition and mood, cognition, and fatigue. More than half of the participants reported pain that affected their daily functioning. Participants with worse pain had greater fatigue and lower total corticospinal excitability (eREC AUC), which accounted for 39.9% of the variance in the frequency of pain, after controlling for age, disability status, and pain medications. Individuals with pain were older, with DMT prescribed less frequently. Groups' disability status, cognitive function, mood, and pain medication prescriptions were not significantly different.

### Pain prevalence, medication use, and fatigue

We found that 55% of participants reported pain, similar to previous estimates.^1,4–6^ As elsewhere, 88% and 70% of all participants had prescriptions for analgesic or neuropathic pain medications.^6,49^ Similar rates of pain medication prescriptions suggest some individuals effectively managed their pain using pharmaceuticals (no pain group). We also found a higher prevalence of fatigue in participants with pain (97%), in line with previous work.^3,4^ A recent longitudinal study showed escalation in pain was associated with baseline fatigue to a greater extent than baseline pain,^4^ suggesting fatigue impairs the ability to function with pain.^3,4^ Cross-sectional work shows that pain and fatigue cluster with depression and cognitive impairment to compromise physical and psychological well-being in an additive and dose-dependent manner.^2^ The lack of differences in mood and cognitive function observed here suggests pain may have distinct relationships with fatigue, mood, and cognition. This warrants further investigation.

### Corticospinal excitability

We found significantly lower total corticospinal excitability (eREC AUC) in participants with pain, but no significant difference in total corticospinal inhibition (iREC AUC). This was contrary to our hypothesis, based on evidence that neuropathic pain is among the most common sources of pain in MS.^1,4–6^ Compared to controls, individuals with neuropathic pain exhibit corticospinal disinhibition,^14,50^ while those with non-neuropathic pain have corticospinal hypoexcitability.^37,51^ The literature shows that most persons with MS experience pain from multiple sources, both neuropathic and non-neuropathic.^5,8,49^ Participants here may therefore have suffered predominantly from non-neuropathic pain,^37,51^ had more pain-associated limb disuse,^52^ or used more medications that target corticospinal excitability (e.g., voltage-gated sodium channel blocking anticonvulsants).^40^ If future work replicates our findings, low corticospinal excitability could be a putative therapeutic target for pain neuromodulation.^13,53^

Others suggest that corticospinal disinhibition is a marker of central sensitization, and a hallmark of all chronic pain—regardless of etiology.^54^ Fatigue, on the other hand, is associated with increased corticospinal inhibition in MS.^33,55,56^ It is therefore possible that greater fatigue, with increased corticospinal inhibition, may have a washout effect on pain-related disinhibition. Future work should disentangle the relationships between, pain, fatigue, and corticospinal excitability and inhibition. A comprehensive set of TMS outcomes (e.g., paired-pulse TMS [intracortical excitability], paired associative stimulation [capacity for neuroplasticity]) may help to establish clearer relationships between symptom clusters and pain pathophysiology.^39,40^

Our findings conflict with other MS research that found no significant difference in RMT or MEP amplitude between participants with and without pain.^15^ Cohen's *d* effect sizes of RMT (*d* = ∼4) and MEP amplitude (*d* = ∼2) in that study were large, suggesting it was underpowered.^15^ But it is also important to acknowledge methodological differences between studies. The previous work collected RMT and resting MEPs, which activate a smaller pool of lower-threshold and slower-conducting cortical neurons than active measures, which stimulate higher-threshold and faster-conducting corticospinal neurons.^40^ Consequently, AMT and active MEPs enable greater characterization of overall corticospinal excitability.^40^ Rather than only measure MEP amplitude at a single low stimulus intensity (120% RMT), we used a range of stimulus intensities (105–155% AMT) to resolve the area under the MEP recruitment curve, which captures the input–output properties of a more diverse range of corticospinal neurons.^39,40^ The previous work therefore may have missed a difference in corticospinal excitability due to methodological constraints.^51^

### Limitations

Although we comprehensively evaluated corticospinal excitability and clinical disease features in a large sample of MS participants, our study had limitations. The median EDSS score was 2 with a narrow IQR and relapsing MS comprised most of the sample, potentially limiting the generalizability of our findings. Our study also had a relatively high drop-out rate—35 of 102 participants did not complete TMS testing because of contraindications, refusal, no-show, or inability to detect MEPs—which could increase selection bias for persons with lower disability and greater function. As well, using the VAS we focused on the functional impact of clinically significant pain on the current day. While we consider this a strength, our method did not delineate pain character or chronicity. We framed our study under the assumption that most participants experienced chronic neuropathic pain^5^; however, the VAS did not distinguish neuropathic pain from other sources. Other instruments can better characterize neuropathic pain and should be used in future work.^57^ We likewise did not explore alternative pain sources like spasticity, musculoskeletal complications of MS, or disease treatments.^8,9^ By not differentiating pain etiology, the VAS may have limited the characterization of pain pathophysiology.

## Conclusions

More than half of MS participants reported pain that affected their daily functioning. Persons with pain had significantly greater fatigue and lower corticospinal excitability, which predicted variance in pain reports. Future work should employ a greater range of TMS outcomes, distinguish neuropathic from other sources of pain, and study a more diverse spectrum of participants. If replicated, these findings suggest that diminished corticospinal excitability in MS-related pain could be a putative therapeutic target for neuromodulation.
